# Calretinin Immunohistochemistery: An Aid in the Diagnosis of Hirschsprung’s Disease

**Published:** 2012

**Authors:** Mehran Hiradfar, Nourieh Sharifi, Mohammad Khajedaluee, Nona Zabolinejad, Shirin Taraz Jamshidi

**Affiliations:** 1 *Department of Pediatric Surgery, Mashhad University of Medical Sciences, Mashhad, Iran*; 2 *Department of Pathology, Mashhad University of Medical Sciences, Mashhad, Iran*; 3 *Department of Community Medicine, Mashhad University of Medical Sciences, Mashhad, Iran*

**Keywords:** Calretinin, Hirschsprung Disease, Immunohistochemistry

## Abstract

**Objective(s):**

Definite diagnosis of Hirschsprung’s disease (HD) is based on histopathological study, but there are limitations associated with standard histology and histochemistry in this regard. The aim of this study was to investigate calretinin immunostaining patterns in both ganglionic and aganglionic HD intestinal specimens and to compare them with control specimens.

**Materials and Methods:**

Specimens included 30 patients with histopathologic diagnosis of HD and 20 patients that underwent colectomy for other reasons (as control group). Eighty paraffin wax blocks of full thickness intestinal specimens (30 blocks of ganglionic segments, 30 blocks of aganglionic segments and 20 blocks of control group) were studied. Calretinin immunoreactivity and pattern of staining for ganglion cells (nuclear and cytoplasmic) and also nerve fibers in different layers of bowel were evaluated in IHC stained slides.

**Results:**

There were positive immunostaining of nerve fibers in the lamina propria, submucosa and muscularis propria in control and patient group. There were also nuclear and cytoplasmic staining of ganglion cells in submucosa and muscularis propria in all specimens of both control group (100%) and ganglionic segments (100%). Calretinin immunoexpression of nerve fibers in muscularis propria of the aganglionic segments was negative in all but two cases (6.7%). This method had sensitivity of 93.3% and specificity of 100% for diagnosis of HD in full thickness specimens of intestinal wall. The positive predictive value was 100% and negative predictive value was 93.8%.

**Conclusion:**

Calretinin immunohistochemistry can be used on suction rectal biopsies as a reliable and adjunctive method to diagnose HD.

## Introduction

Hirschsprung’s disease (HD, aganglionic megacolon) is a common cause of congenital intestinal pseudo-obstruction occurring in 1 out of 5000 live births. It is characterized by the absence of intramural ganglion cells and the presence of excessive numbers of cholinergic nerve fibers (1, 2). Although anorectal manometry or radiologic studies are used to establish the diagnosis of HD, the definite diagnosis is made by histologic demonstration of agangliosis in both the myenteric (Auerbach) and submucous (Meissner) plexuses (3-5). Identification of ganglion cells in the myenteric plexus is easier, but needs a full-thickness rectal biopsy that requires a surgical procedure under general anesthesia and also is associated with some morbidity and technical difficulties. Rectal suction biopsy is increasingly becoming the procedure of choice for obtaining specimens for the initial diagnosis of HD (6). However, there are limitations to this technique such as morphologic immaturity of ganglion cells particularly in neonates and infants and also the need for evaluation of many sections (more than 50) before a biopsy can be interpreted as negative for ganglion cells which is a time consuming process (2). For these reasons a number of ancillary methods have been introduced to facilitate the diagnosis. The most widely applied technique is acetylcholinesterase histochemistry (AChE) (7-10). AChE stain shows an increase in coarse cholinergic nerve fibers in the muscularis mucosae, sometimes extending into the lamina propria. This technique requires frozen tissue samples and, there are false-negative results mostly due to the young age of patients (2, 8).

The aim of our study was to investigate calretinin immunostaining patterns in both ganglionic and aganglionic HD intestinal specimens and to compare them with control specimens. This study was done for the first time in Iran.

## Materials and Methods

This is a retrospective study on specimens from patients with histopathologic diagnosis of HD that underwent radical operation in Dr Sheikh Children Hospital, Mashhad University of Medical Sciences between 2008 and 2010. 

For each patient one full thickness paraffin block from ganglionic and another block from aganglionic segment were retrieved.

In addition, 20 colon specimens (in which the diagnosis of HD was excluded based on H&E identification of ganglion cells) matched for age and sex, were used as control group. 

In total, 80 paraffin blocks, previously fixed in 10% buffered formaline, were examined and processed. These comprised 30 blocks from the aganglionic zone, 30 from the ganglionic zone and 20 blocks from control group.

Immunostaining was performed on paraffin embedded archival tissues following the Avidin –Biotin peroxidase technique. In brief, sections measuring 3-5 µm thick were cut, air dried for 15 min, heat-fixed at 42 ˚C and then air-dried overnight at room temperature. After deparaffinization with xylene, endogenous peroxidase activity was eliminated by treating the slides with AOH/HO for 30 min at room temperature. Then the slides were incubated with the diluted primary antibody (Clone: DAK- Calret 1, monoclonal mouse anti- human, Dako, Denmark,1/100 dilution) at 40 ˚C in a humidified chamber for 60 min. Biotinylated antimouse IgG and avidin-biotin peroxidase complex were added in sequence. The sections were then incubated with DAB for 10 min for visualization of the peroxidase reaction. After being washed in water for few minutes, the sections were counter stained with hematoxylin, dehydrated in alcohol, cleared in xylen and mounted.

Calretinin immunoreactivity and pattern of staining for ganglion cells (nuclear and cytoplasmic) and also nerve fibers in different layers of bowel (lamina propria, muscularis mucosa, submucosa and muscularis propria) were evaluated in IHC stained slides. Epithelium of plural tissue was selected as positive control, and antibody was omitted from staining process for negative control. Two pathologists independently reviewed the hematoxylin and eosin and immunohistochemichal stained slides and agreed on diagnoses by consensus.

Statistical calculations were carried out using SPSS version 11.5. For description of results in each group, methods of descriptive analysis such as central tendency, dispersion and frequency distribution were used as tables. 

## Results

Patient’s age varied from 2 days to 12 years (mean, 17±4 months) and male-to female ratio was 2.75.

**Table 1:** Prevalence of calretinin expression in nerve fibers of different layers of intestine in patients with HD^*^ and in control group

	Patients (30)	Control group (20)	
Layers of intestine	n (%)	Ganglionic bowels n (%)	Aganglionic bowels n (%)
Lamina propria	19 (95)	21 (70)	0 (0)
Muscularis mucosa	20 (100)	25 (83)	0 (0)
Submucosa	20 (100)	30 (100)	0 (0)
Muscularis propria	20 (100)	30 (100)	2 (6.7%)

*Hirschsprung disease

**Figure 1:**
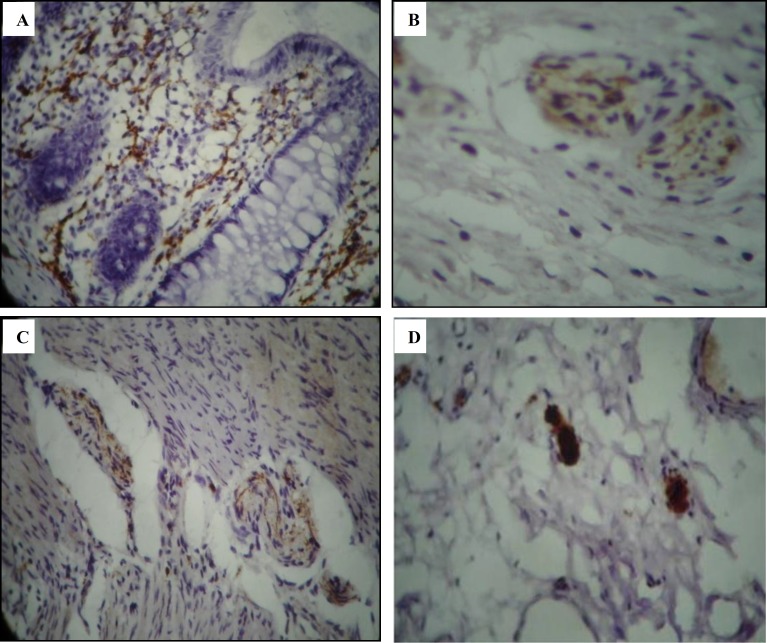
Positive calretinin immunostainig of nerve fibers in lamina propria (A), submucosa (B), and muscularis mucosa (C). Nuclear and cytoplasmic calretinin immunostainig of ganglion cells (D)

**Table 2:** Prevalence of calretinin expression in nerve fibers of different layers of intestine in patients with HD considering to extent of disease

Layers of intestine	Types of HD	
	Short segment and classic	Long segment, extended and total colonic
	Ganglionic aganglionic n (%) n (%)	Ganglionic aganglionic n (%) n (%)
Lamina propria	14 (70%) 0 (0)	7 (70) 0 (0)
Muscularis mucosa	16 (80%) 0 (0)	9 (90) 0 (0)
Submucosa	20 (100%) 0 (0)	10 (100) 0 (0)
Muscularis propria	20 (100%) 2 (6.7%)	10 (100) 0 (0)

**Figure 2:**
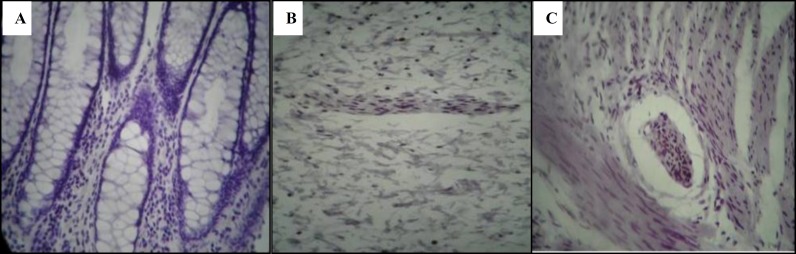
Total absence of staining after calretinin immunohistochemistry in the aganlionic segment in lamina propria (A), submucosa (B) and muscularis propria (C)

There were 17 classic cases (56.7%), 7 long segment cases (23.3%), 3 short segment cases (10%), 2 extended cases (6.7%), and 1 case of total colonic aganglionosis (3.3%).

There were positive immunostaining of nerve fibers in the lamina propria, muscularis mucosa, submucosa and muscularis propria in control and patient groups (Figure1 & Table 1). This immunoexpression was present in different types of HD (with different extents of aganglionosis) (Table 2).

There were also nuclear and cytoplasmic staining of ganglion cells in submucosa and muscularis propria in all specimens of both control group (100%) and ganglionic bowels (100%) (Figure 1). The immunostaining of ganglion cells was specific, and no other cells were stained.

**Figure 3:**
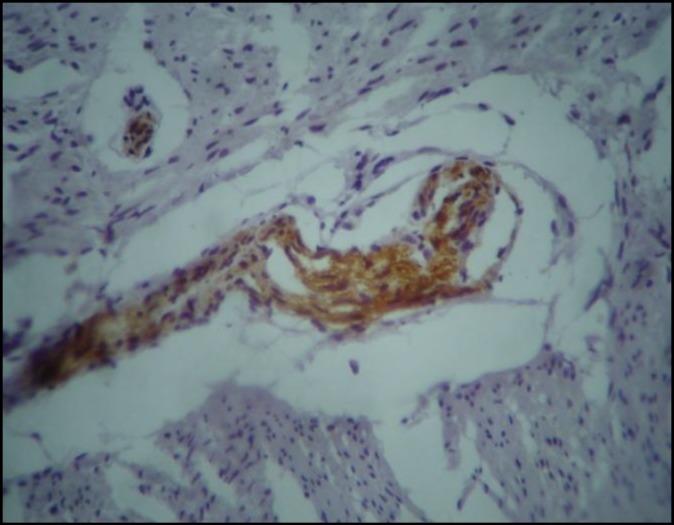
False positive immunostaining of nerve fibers in muscularis propria in aganglionic segment

Calretinin immunoexpression was negative in all but two cases (6.7%) of aganglionic segments. One case was a 12-month-old boy with classic HD with a pattern of immunostaining in the ganglionic bowel similar to control group. We found positive immunostaining only in hypertrophic nerve bundles of muscularis propria in the ganglionic segment (Figure 3). Another case was a19-month-old girl with short segment HD that showed normal immunostaining in the ganglionic part, and positive immunostaining only in large nerves of muscularis propria of the aganglionic segment. So, this method had sensitivity of 93.3% and specificity of 100% for diagnosis of HD in full thickness specimens of intestinal wall. The positive predictive value was 100% and negative predictive value was 93.8%. 

## Discussion

Diagnosis of HD can be a stressful practice, particularly for pathologists who infrequently encounter the condition. Limitations associated with a H&E-based approach to the diagnosis of HD, with or without AChE histochemistry, urged us to use another reliable diagnostic method such as immunohistochemistry to facilitate diagnosis (9).

Several immunohistochemical markers have been introduced, but most of them have limitations for use in daily practice (10).

Calretinin is a vitamin D-dependent calcium-binding protein involved in the physiological buffering of excess cytosolic calcium ions; calcium transport and protection against calcium ion overload (11).

McConalgue *et al* in 1994 showed that calretinin immunoreactivity reveals different neuronal populations in the large intestine of guinea-pig and after that this property has been shown for all ganglion cells and nerve fibers (12). In 2004 Barshack *et al* investigated calretinin immunorectivity in ten large bowels, full thickness specimens (a total 54 paraffin wax blocks) from patients with classic rectosigmoid HD. Calretinin was not expressed in aganglionic segments of HD and associated nerve fibers, whereas both ganglion cells and nerve fibers were immunopositive in ganglionic HD segments and in normal colons. The transitional zone showed a broad spectrum of histomorphological and immunohistochemical patterns of calretinin expression (13).

In another recent study by Kapur *et al* in 2009 multiple observers independently reviewed calretinin IHC and AChE sections of suction biopsies from 14 HD and 17 controls. There were 2 misdiagnoses and more disagreements in the interpretation of AChE-stained sections, but calretinin IHC yielded no misdiagnoses or discrepancies. In Kapur’s study, similar to ours, both nuclear and cytoplasmic immunoreactivity was present in ganglion cells and also there was positive staining of nerve fibers of lamina propria, submucosa and muscularis mucosa of ganglionic biopsies. They concluded that calretinin IHC can be a superior, alternative to AChE as an adjunctive diagnostic method for evaluating suction rectal biopsies for HD (14). It is interesting to note that Kapur *et al* found some immunoreactive axons in large nerves of the muscularis mucosa similar to what we found in 2 of our cases in large nerves of the muscularis propria. They stated that immunostaining in large submucosal nerves (>20 µ) in HD consists of puctate evenly spaced axons like the calretinin immunoreactivity in normal serosal nerves and is distinctly different from confluent granular staining of small-caliber nerves (< 15 µ) in the normal superficial submucosa (14). 

In another study in 2009, Guinard-Samuel *et al* evaluated the calretinin immunostaining as a primary diagnostic tool on a large series of suction rectal biopsies .They retrieved 131 biopsies carried out for suspicion of HD in children and infants to compare the accuracy of calretinin immunohistochemistry with the standard method (histology and acetylcholinesterase staining) (15).

In their study, calretinin immunohistochemistry enabled the diagnosis of all HD diagnosed by the standard technique, except for one patient who had a weak positive immunostaining in some nerve fibers(false negative case). It is important to note that 12 additional cases initially considered as suspicious for HD using the standard technique were accurately diagnosed by calretinin immunohistochemistry. They suggested that calretinin might be more accurate than acetylcholinesterase in detecting aganglionosis (15).

So far 3 previous studies have been published to evaluate calretinin IHC in diagnosis of HD (13-15). The number of cases were fewer than ours in two of these studies (9, 13, 14). Only in Guinard-Samuel’s study a large series of rectal suction biopsies were used to assess the diagnostic value of calretinin IHC in the diagnosis of HD (15).We evaluated different layers of bowel (lamina propria, muscularis mucosa and muscularis propria) separately, and explored the results in cases with different extents of HD (although the number of cases in each type of HD were limited), which were not addressed in the previous studies.

Guinard-Samuel *et al* compared the accuracy of this technique with AChE staining (15). Although AChE staining has been introduced as an ancillary method in the diagnosis of HD, it has many limitations. Part of these limitations may be related to difficulties in performing and interpreting it even for the pathologists experienced in diagnosing HD (14, 16). Furthermore, presence of false-nagative results due to the young age of patients or long segments of aganglionosis can also limit its application in our daily practice (8, 15). On the other hand immunohistochemistry is based on paraffin sections and its interpretation which is based on negative or positive results is much easier (14, 15).

Nowadays, full thickness specimens are being replaced by suction or mucosal rectal biopsies in the diagnosis of HD. Interpretation is more difficult in these specimens. By facilitating the diagnosis, IHC staining may encourage more and more pathologists and surgeons to use suction biopsies instead of using the more invasive diagnostic technique of using full thickness specimens. 

## Conclusions

As there were no false- negative or false-positive results based on calretinin immunostaining in submucosa in our study, it can be used on suction rectal biopsies as a reliable and adjunctive method to diagnose HD.
